# Osteogenic Enhancement Between Icariin and Bone Morphogenetic Protein 2: A Potential Osteogenic Compound for Bone Tissue Engineering

**DOI:** 10.3389/fphar.2019.00201

**Published:** 2019-03-12

**Authors:** Xin Zhang, Xingnan Lin, Tie Liu, Liquan Deng, Yuanliang Huang, Yuelian Liu

**Affiliations:** ^1^Department of Periodontics, Hospital/School of Stomatology, Zhejiang University, Hangzhou, China; ^2^ACTA, Department of Oral Implantology and Prosthetic Dentistry, Research Institute, University of Amsterdam and VU University Amsterdam, Gustav Mahlerlaan, Netherlands; ^3^Department of Orthodontics, Nanjing Stomatological Hospital, Nanjing University Medical School, Nanjing, China; ^4^Department of Oral Implantology, Hospital/School of Stomatology, Zhejiang University, Hangzhou, China; ^5^School of Stomatology, Zhejiang Chinese Medical University, Hangzhou Dental Hospital, Hangzhou, China; ^6^Department of Dentistry, Shanghai East Hospital Affiliated to Tongji University, Shanghai, China

**Keywords:** biomimetic calcium phosphate, MC3T3-E1, critical-sized bone defect, Icariin, bone morphogenetic protein 2

## Abstract

Icariin, a typical flavonol glycoside, is the main active component of Herba Epimedii, which was used to cure bone-related diseases in China for centuries. It has been reported that Icariin can be delivered locally by biomaterials and it has an osteogenic potential for bone tissue engineering. Biomimetic calcium phosphate (BioCaP) bone substitute is a novel drug delivery carrier system. Our study aimed to evaluate the osteogenic potential when Icariin was internally incorporated into the BioCaP granules. The BioCaP combined with Icariin and bone morphogenetic protein 2 (BMP-2) was investigated *in vitro* using an MC3T3-E1 cell line. We also investigated its efficacy to repair 8 mm diameter critical size bone defects in the skull of SD male rats. BioCaP was fabricated according to a well-established biomimetic mineralization process. *In vitro*, the effects of BioCaP alone or BioCaP with Icariin and/or BMP-2 on cell proliferation and osteogenic differentiation of MC3T3-E1 cells were systematically evaluated. *In vivo*, BioCaP alone or BioCaP with Icariin and/or BMP-2 were used to study the bone formation in a critical-sized bone defect created in a rat skull. Samples were retrieved for Micro-CT and histological analysis 12 weeks after surgery. The results indicated that BioCaP with or without the incorporation of Icariin had a positive effect on the osteogenic differentiation of MC3T3-E1. BioCaP with Icariin had better osteogenic efficiency, but had no influence on cell proliferation. BioCap + Icariin + BMP-2 showed better osteogenic potential compared with BioCaP with BMP-2 alone. The protein and mRNA expression of alkaline phosphatase and osteocalcin and mineralization were higher as well. *In vivo*, BioCaP incorporate internally with both Icariin and BMP-2 induced significantly more newly formed bone than the control group and BioCaP with either Icariin or BMP-2 did. Micro-CT analysis revealed that no significant differences were found between the bone mineral density induced by BioCaP with icariin and that induced by BioCaP with BMP-2. Therefore, co-administration of Icariin and BMP-2 was helpful for bone tissue engineering.

## Introduction

Bone defects are quite common. They result from inflammatory disease, tumor, trauma, or anatomical and congenital disorders. When the defects do not heal by themselves, grafting with a bone or a bone substitute is needed to achieve a good functional and a great aesthetic restoration (Lewandrowski et al., 2000; Hamann et al., 2013). The gold standard for bone reconstruction is the autologous bone graft (van den Bergh et al., 1998), which has many advantages because of its natural osteoinductivity. However, there are also disadvantages in using a bone transplant, such as the limited availability of bone, morbidity of the donor site and the risk of infection. The application of allografts, xenografts, or biosynthetic substitutes eliminates these disadvantages of using an autologous bone graft (Meijer et al., 2007).

Biosynthetic substitutes, such as biomaterials based calcium phosphate (CaP) have been used successfully as a graft because of their good biocompatibility, osteoconductivity, and the chemical composition, which resembles the composition of the natural bone matrix (Bauer and Muschler, 2000; Kurashina et al., 2002; Habraken et al., 2007; Galea et al., 2008; Dorozhkin, 2010). Recently, biomimetic calcium phosphate (BioCaP) based materials have received an increased interest because of their capacity to carry and deliver bioactive agents without compromising their bioactivity (Liu et al., 2004, 2010; Panzavolta et al., 2010; Buschmann et al., 2012; Tanase et al., 2012). Biomimetic materials are capable of eliciting specific cellular responses and directing new tissue formation (Shin et al., 2003). Liu et al. (2013) recently refined the use of a BioCaP bone substitute. Protein and CaP were precipitated together to form BioCaP granules in which protein was incorporated in the center of the granules to form an internal depot. Previous studies have shown that the volume density of the bone, bone marrow, and the blood vessels were significantly higher when granules with BMP-2 incorporated internally were used.

Recently, we have found that Icariin is a strong bone anabolic agent, which is comparable with BMP-2 in enhancing the proliferation and osteogenic differentiation of MC3T3-E1 cells (Zhang et al., 2014b). Icariin is a small molecule, which is the main pharmacological component of Herba Epimedii, a centuries-old traditional herb medicine (Zhang et al., 2014a). Previous studies have shown that Icariin can increase osteogenic differentiation (Chen et al., 2005; Ma et al., 2011; Zheng et al., 2012) and inhibit osteoclastic formation (Chen et al., 2007; Hsieh et al., 2011). When loaded into a CaP cement scaffold (Zhao et al., 2010), a β-tricalcium phosphate ceramic (Zhang et al., 2011) or a chitosan/hydroxyapatite (Wu et al., 2009), it could promote bone regeneration. In addition, a 24-month clinical trial using a randomized double-blind with a placebo indicated that a daily dose of 60 mg Icariin with 15 mg daidzein, and 3 mg genistein has beneficial effects in preventing bone loss in postmenopausal women without resulting in a detectable hyperplasia effect on the endometrium (Zhang et al., 2007).

The application of BMP-2, which is approved by the United States Food and Drug Administration, have been widely used to effectively promote bone formation (Agrawal and Sinha, 2017). However, a high dose of BMP-2 can cause unwanted calcification (Zebboudj et al., 2003), abnormal bone resorption (Zara et al., 2011), an unexpected bone formation in the ectopic area (Uludag et al., 1999), and it may stimulate cancer cell growth (Kleeff et al., 1999). The easiest way to reduce the problems of safety and supply is to reduce the dose of BMP-2 that is used. Therefore, it is necessary to look for effective biomolecules that can promote the osteogenic bioactivity of BMP-2. Following the results of our previous study, we have made an *in vitro* study of the results of combining Icariin and BMP-2.

Our previous study has demonstrated that Icariin can promote the osteogenic efficiency of BMP-2 in MC3T3-E1 cells, which are a precursor of functionalized osteoblast (unpublished data). To further enhance and control the delivery of BMP-2 and Icariin, these two biomolecules would be incorporated into the interior of bone substitute. In this present study, we prepared BioCaP modified with Icariin and BMP-2. We investigated the biocompatibility and osteogenic efficiency of the scaffold as an effective controlled delivery system.

## Materials and Methods

### Fabrication of the Biomimetic Calcium Phosphate (BioCaP) Bone Substitute

Briefly, a supersaturated CaP solution (200 mM HCl, 20 mM CaCl_2_⋅2H_2_0, 680 mM NaCl, and 10 mM Na_2_HPO_4_) with or without agents [Icariin (≥99%, Tauto Biotech, Shanghai, China) and/or BMP-2 (R&D system, Minneapolis, MN, United States)] (the concentration of the two agents *in vitro* and *in vivo*, respectively, see Table 1) buffered by TRIS (250 mM) to a PH of 7.4. This solution was incubated in a shaking water bath (50 agitations/minute) at 37°C. Rapid precipitation appeared at a pH of 6.25. Protein was added to this CaP solution and co-precipitated (incorporated) into the interior of BioCaP (viz., the internal depot of protein). After 24 h of incubation, the precipitate was retrieved, gently washed by Milli-Q water, filtered, and compressed to form a tablet (diameter: 5 mm; thickness: 0.4 mm) using a vacuum exhaust filtering method with a vacuum filter (0.22-lm pore, Corning, NY, United States) and an air pump. After drying at room temperature, BioCaP tablets were ground and filtered using metallic mesh filters to obtain granules with a size of 0.3–0.6 mm. For sterilization, all the solutions were filtered with the vacuum filter (0.22-μm pore) before co-precipitation. All the procedures were performed under aseptic conditions.

**Table 1:** Concentration of agents introduced into the CaP solutions before buffering.

	Concentration of agent	
	*In vitro*	*In vivo*
BioCaP + Icariin	5 mg/L	10 mg/L
BioCaP + BMP-2	0.5 mg/L	0.5 mg/L
BioCaP + Icariin +BMP-2	5 + 0.5 mg/L	10 + 0.5 mg/L

### *In vitro* Experiment

#### Experiment Groups

To test the osteogenic efficiency of the BioCaP incorporated with Icariin and BMP-2, the MC3T3-E1 cell line was treated in the following experimental groups:

1. Cultural medium (C);
2. BioCaP granules alone (Bio);
3. BioCaP granules with an internal depot of Icariin (Bio + I, experimental)
4. BioCaP granules with an internal depot of BMP-2 (Bio + B, experimental)
5. BioCaP granules with an internal depot of Icariin and BMP-2 (Bio + I + B, experimental)

#### Culture of MC3T3-E1 With BioCaP

MC3T3-E1 Cell (Chinese Academy of Sciences, Shanghai, China) suspensions were plated at 4 × 10^5^ cells/well in six well plated for cell proliferation, alkaline phosphatase (ALP) activity assay, osteocalcin (OCN) expression, alizarin red staining, and osteogenic gene expression. After 24 h incubation, 12–15 mg BioCaP alone or BioCaP with Icariin or/and BMP-2 was seeded at each well.

#### Cell Proliferation Assay

To investigate the proliferation of MC3T3-E1 cells in response to BioCaP alone or BioCaP with Icariin or/and BMP-2, the number of cells was determined after stimulation for 1, 4, and 7 days using Cell Counting Kit-8 (Donjindo, Kumamto, Japan). Cells were seeded in 96-well plates. After refreshing the medium 24 h after seeding, 100 μL of culture medium with different groups was added to each well. The treatment medium was refreshed every 3 days. After 1, 4, and 7 days incubation, the treatment medium was replaced with 100 μL CCK-8 working solution according to the manufacturer’s instruction. The OD (optical density) values were measured at 450 nm after 40 min incubation.

#### ALP Activity Assay

To determine the early differentiation of preosteoblasts stimulated by BioCaP alone or BioCaP with Icariin or/and BMP-2, the ALP activity and the protein content were measured after treatment on days 1, 4, and 7. ALP activity in the cell lysates was determined using the LabAssay ALP colorimetric assay kit (Wako, Osaka, Japan). The cell number was estimated by determining total protein content measured at 570 nm using a commercial bicinchoninic acid (BCA) Protein Assay kit (Beyotime, Shanghai, China). The values representing ALP activity were expressed as mmol p-NP/mg total protein.

#### OCN Expression Assay

To assess the terminal differentiation of MC3T3-E1 cells stimulated by BioCaP alone or BioCaP with Icariin or/and BMP-2, OCN secreted into the cell culture medium was determined. The cell supernatants were collected on days 4, 7, and 10 and were centrifuged (10,000 rpm, 4°C, 5 min) before detection. The supernatant OCN concentration was determined using the ELISA mouse OCN EIA kit (Biomedical Technologies, Stoughton, MA, United States).

#### Extracellular Matrix Mineralization

We compared the mineralization of the MC3T3-E1 cells stimulated by BioCaP alone or by BioCaP with Icariin and/or BMP-2. Triplicate cell cultures were prepared in the same way as described previously and then treated with a mineralizing medium [10% fetal bovine serum (FBS) (Hangzhou Tianhang Bio-technology Co., Hangzhou, China), 50 mg/mL L-ascorbic acid-2-phosphate (AsAP), and 10 mM β-glycerophosphate (β-GP) (Sigma-Aldrich, St. Louis, MO, United States)] containing. The medium was replaced every 3 days. After 3, 4, and 5 weeks, the mineralized nodules were determined by alizarin red (Sigma-Aldrich, St. Louis, MO, United States) staining. The culture plates were photographed with NIS-Elements F2.20 (Nikon Eclipse 80i, Tokyo, Japan), and the calcified area was measured using an Image-Pro Plus 6.0 analysis. After being photographed, the mineralized nodules and the release calcium-bound alizarin red S was dissolved using 10% cetylpyridinium chloride (CPC, Sigma-Aldrich LLC, GER). The colorimetric absorbance at 560 nm was then measured.

#### Real-Time PCR Quantification of Gene Expression

The effects of BioCaP alone and BioCaP with Icariin and/or BMP-2 on stimulating the osteogenic gene expression were also examined by quantitative RT-PCR. The messenger ribonucleic acid (mRNA) expression of ALP, BMP2, OCN, collagen-I (Col I), and runt-related transcription factor 2 (Runx-2) was measured after 1, 4, and 7 days of osteogenic induction. The total RNA was extracted from the cells using the RNeasy Mini Kit and RNase-Free DNase Set (Qiagen sample and assay technologies, Germany). Single stranded complementary deoxyribonucleic acid (cDNA) was synthesized from total ribonucleic acid (RNA) with a Primescrip^TM^ RT Reagent Kit (Takara Biotechnology, Dalian, China). A real time polymerase chain reaction (PCR) was performed using 1 μl of cDNA product in a 25 μl reaction volume with Mastercycler ep realplex Real Time PCR System (Eppendorf, Germany). SYBR^®^ Premix Ex TaqTM II (Takara Biotechnology, Dalian, China), specific primers (see Table 2), and 1 μl of cDNA were used in each PCR reaction (95°C for 30 s, 40 cycles of denaturation at 95°C for 5 s, and annealing and extension at 60°C for 30 s). The sense and antisense primers (see Table 2) were designed with the Primer Express 3.0 based on published mouse cDNA sequences. β-actin was used as an internal control gene. All real time PCR reactions were performed in triplicate. The results were calculated using the comparative threshold cycle (DDCT) method – after calibration with β-actin expression – and are presented as fold increase relative to the unstimulated control.

**Table 2:** Primer sequences for real-time quantitative polymerase chain reaction analysis of the gene expression.

Gene	Accession No.	Primers (F = forward; R = reverse)
ALP	NM_007431	F: 5′- TGCCTACTTGTGTGGCGTGAA -3′;
		R: 5′- TCACCCGAGTGGTAGTCACAATG -3′
BMP-2	NM_007553	5′-AAGAGACATGTGAGGATTAGCAGGT-3′ and
		5′-GCTTCCGCTGTTTGTGTTTG-3′
Collagen I	NM_007742	F: 5′- ATGCCGCGACCTCAAGATG -3′;
		R: 5′- TGAGGCACAGACGGCTGAGTA -3′
Osteocalcin (OCN)	NM_007541	F: 5′- AGCAGCTTGGCCCAGACCTA -3′;
		R: 5′- TAGCGCCGGAGTCTGTTCACTAC -3′
Runx2	NM_009820	F: 5′- CACTGGCGGTGCAACAAGA -3′;
		R: 5′- TTTCATAACAGCGGAGGCATTTC -3′
β-actin	NM_007393	F: 5′- AGGAGCAATGATCTTGATCTT -3′;
		R: 5′- TGCCAACACAGTGCTGTCT -3′

#### Statistical Analysis

The results were first assessed using a Kolmogorov–Smirnov test to ensure normality and homogeneity of variance. As the data were normally distributed, statistical comparisons of the results obtained with BioCaP alone or BioCaP with Icariin and/or BMP-2 were made with a one-way analysis of variance. *Post hoc* comparisons were made using Bonferroni corrections. The level of significance was set at *p* < 0.05. The statistics software package for social science (SPSS) (version 17, SPSS Inc., Chicago, IL, United States) for a Windows computer system was employed for the statistical analysis.

### *In vivo* Experiment

#### Experiment Groups

Surgical procedures were performed on 8 weeks old male SD rats. The animal experiments were carried out in accordance with the Guide for the Care and Use of Laboratory Animals and were approved by the Ethics Committee of Zhejiang Chinese Medical University, China. A critical-sized bone defect was treated in four groups with 6 animals in each group as follows:

1. BioCaP granules with neither Icariin nor BMP-2 (BioCaP, negative control);
2. BioCaP granules with an internal depot of BMP-2 (BioCaP int. BMP-2, experimental);
3. BioCaP granules with an internal depot of Icariin (BioCaP int. Icariin, experimental);
4. BioCaP granules with an internal depot of icariin and BMP-2 (BioCaP int. Icariin and BMP-2, experimental);

#### Surgical Procedure

**FIGURE 1:**
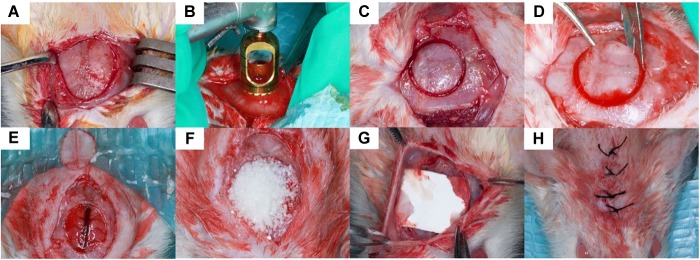
Surgical procedure. 8 mm critical sized defect was created was made on the scalp of the rats **(A–D)**, the granules were randomly implanted into the defects and covered by Bio-gide membranes **(E–G)**. The incision was closed in layers **(H)**.

The animals were anesthetized by an intraperitoneal injection of Sumianxin II (0.5 ml/kg) (Military Veterinary Institute, Quartermaster University of PLA, Changchun, China). A 2 cm sagittal incision was made on the scalp and the calvarium was exposed by a blunt dissection (Figure 1A). One critical sized bone defect was created with an 8-mm diameter trephine bur (Hu-Friedy, United States) (Figure 1B–D) being careful not to perforate the dura mater (Takagi and Urist, 1982) and superior sagittal sinus (Figure 1E). The granules of every experimental group were randomly implanted into the critical sized calvarial defects (Figure 1F). The defects were then covered with Bio-gide membranes (Figure 1G). The incision was closed in layers with 4-0 sutures (Figure 1H). The rats were able to function normally after this procedure.

#### Micro-CT Evaluation

**FIGURE 2:**
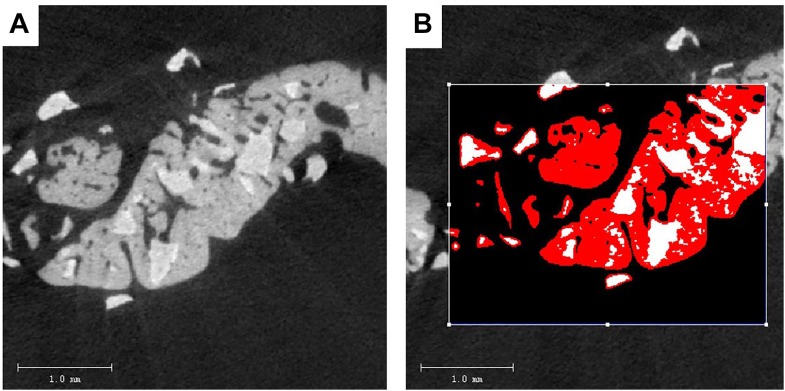
Two-dimensional (2D) images of BioCaP and bone by Micro-CT **(A)**. Bone (red) and BioCaP (white) were separated by the analysis software **(B)**.

Specimens with 2 to 3 mm surrounding the critical sized bone defect were fixed in a 10% neutral buffered formalin solution. The undecalcified specimens of 30 rats were dehydrated in concentrations of ethanol increasing from 70 to 100% and finally embedded in methyl methacrylate (MMA) (Zou et al., 2011). Three-dimensional reconstructions of the embedded specimens were scanned using a high-resolution micro-CT system (μCT 40, Scanco Medical AG, Bassersdorf, Switzerland). The specimens were placed vertically in a polyetherimide cylindrical holder and scanned at 70 kV source voltage, 113 μA current and a 18 μm isotropic voxel size. The gray values of each specimen which depends on the radiopacity of the scanned material, were converted into the degree of mineralization with the analysis software (Scanco Medical AG). Newly formed bone and BioCaP can easily be seen apart because the mineralization of the BioCaP is significantly whiter than that of the bone (Figure 2) As in the previous study, the novel “onion-peeling” algorithm (Scanco Medical AG) was used to distinguish between the newly formed bone deposited on the BioCaP and the BioCaP itself and specific threshold settings. By using this approach, the micro-CT results were comparable with histomorphometrical ones (Schulten et al., 2013). Briefly, a low threshold of 560 mg hydroxyapatite (HA)/cm^3^ was used to discriminate bone tissue from connective tissue and bone marrow. The gray values were scaled from 1 to 1000 and the threshold was set at 200 to distinguish BioCaP from bone tissue. These two thresholds were calculated by averaging the thresholds resolved in three slices of three samples by two individual researchers. Micro-CT measurements included bone volume (BV), bone density (BV/TV), material volume (MV), and bone mineral density (BMD).

#### Histological Procedures

The specimens were sawn into 10–12 slices vertical to the surface of the defects. The slices were subsequently polished to a final thickness of about 600 μm. They were then stained on the surface with McNeal’s Tetrachrome, basic Fuchsine and Toluidine Blue (Wu et al., 2011) and examined with a light microscope with a digital camera (Leica, Wetzlar, Germany).

#### Histomorphometric Analysis

In addition to a subjective histological description, three sections chosen at random from each sample were used for the quantitative histomorphometric analysis. The volume density of newly formed bone was measured using an image analysis system (image Pro 5.0, Media Cybernetic, Sliver Springs, MD, United States) and reported as a percentage of the whole calvarium defect area (Zou et al., 2011).

#### Statistical Characterization

The Kolmogorow–Smirnov test was used to analyze the data normality. All data are presented as the mean value and standard deviation (SD). Using the software SPSS 16.0 (SPSS Science), the statistical significance was assessed with a Turkey’s *post-hoc* test of a one way analysis of variance (ANOVA). The significance level was set at *p* < 0.05.

## Results

### *In vitro* Experiment

#### Cell Proliferation Assay

**FIGURE 3:**
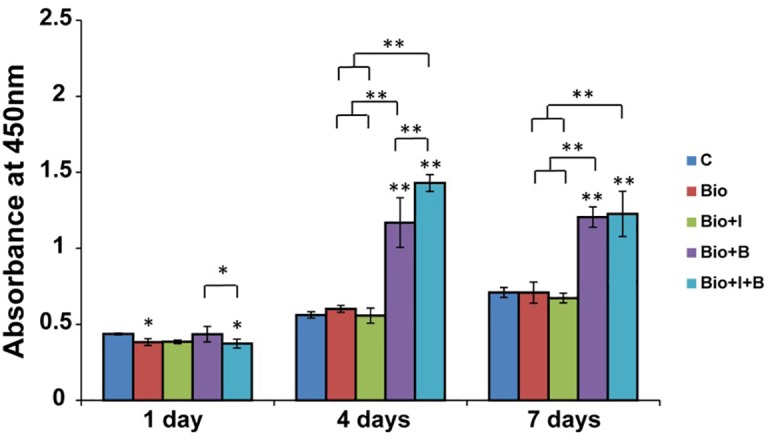
The proliferation of MC3T3-E1 cells stimulated by BioCaP alone or BioCaP with Icariin or/and BMP-2. The absorbance was measured on days 1, 4, and 7. Mean values (*n* = 3 samples per group) were represented together with the standard deviation. Error bars denote the standard deviation. ^∗^*P* < 0.05, ^∗∗^*P* < 0.01, ^∗∗^without lines means versus control (C). C, Control; BioCaP, biomimetic calcium phosphate; I, Icariin; B, BMP-2.

BioCaP with or without Icariin did not affect cell proliferation of MC3T3-E1 at all three times compared with control group. BioCaP with Icariin did not improve the cell proliferation compared with using BioCaP granules alone. However, BioCaP either with BMP-2 or with Icariin and BMP-2 both increased the cell proliferation significantly after 4 and 7 days compared with the control group but not on the first day. BioCaP incorporated with both Icariin and BMP-2 had a higher cell proliferation than BioCaP with BMP-2 alone on day 4 (Figure 3).

#### ALP Activity Assay

**FIGURE 4:**
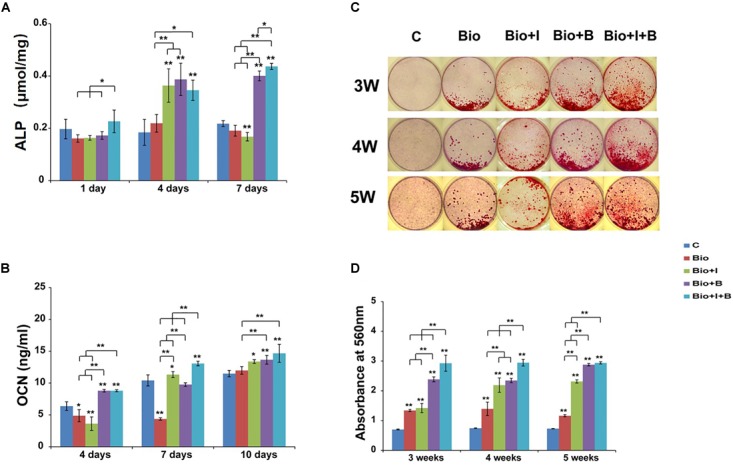
Osteogenic differentiation results of MC3T3-E1 cells after treatment with BioCaP alone or BioCaP with Icariin or/and BMP-2. **(A)** The ALP activities were determined by colorimetric assay on days 1, 4, and 7. **(B)** The OCN expressions on days 4, 7, and 10. **(C)** Macroscopic images of alzarin red stained 3, 4, and 5 weeks after five groups treatment. **(D)** The mineralization results on 3, 4, and 5 weeks. Mean values (*n* = 3 samples per group) were represented together with the standard deviation normalized by total cellular protein. Error bars denote the standard deviation. ^∗^*P* < 0.05, ^∗∗^*P* < 0.01, ^∗∗^without lines means versus control (C). C, Control; BioCaP, biomimetic calcium phosphate; I, Icariin; B, BMP-2.

After 4 and 7 days treatment, BioCaP with both of Icariin and BMP-2 had higher ALP activity than BioCaP granules alone (1.6 times and 2.3 times) and the control group (1.9 times and 2 times). Moreover, with the addition of Icariin, BioCaP with both of two agents produced more ALP than BioCaP with BMP-2 alone on day 1 (1.3 times) and day 7 (1.1 times). Also, higher ALP activity was produced by BioCaP with Icariin than nontreated control (2 times) and the BioCaP alone (1.7 times) group on day 4 (Figure 4A).

#### OCN Expression Assay

BioCaP alone produced less OCN expression than the control group after 4 and 7 days. Interestingly, when BioCaP is incorporated with Icariin, the OCN secretion is 1.1 times greater after 7 days and 1.2 times greater after 10 days compared with the control group. BioCaP incorporated with Icariin and BMP-2 had higher OCN expression than the control group, 1.4, 1.4, and 1.7 times, respectively. (Figure 4B). Seven days after treatment, BioCaP incorporated with Icariin and BMP-2 produced 1.4 times more OCN than BioCaP with BMP-2 but there was no statistically significant difference after 4 and 10 days.

#### Extracellular Matrix Mineralization

On all three time points, all the other 4 groups had a significant higher mineralization compared with the control group. BioCaP with Icariin showed far more areas of mineralized nodules compared with the BioCaP granules alone on the later days, 1.6 times greater after 4 weeks and 2 times greater after 5 weeks. Incorporating with Icariin, BioCaP with BMP-2 can have more mineralization on 3 weeks (1.2 times) and 4 weeks (1.3 times). 5 weeks after treatment, there were no significant differences in mineralization between BioCaP with Icariin and BMP-2 and BioCaP with BMP-2 (Figure 4C,D).

#### Real-Time PCR Quantification of Gene Expression

**FIGURE 5:**
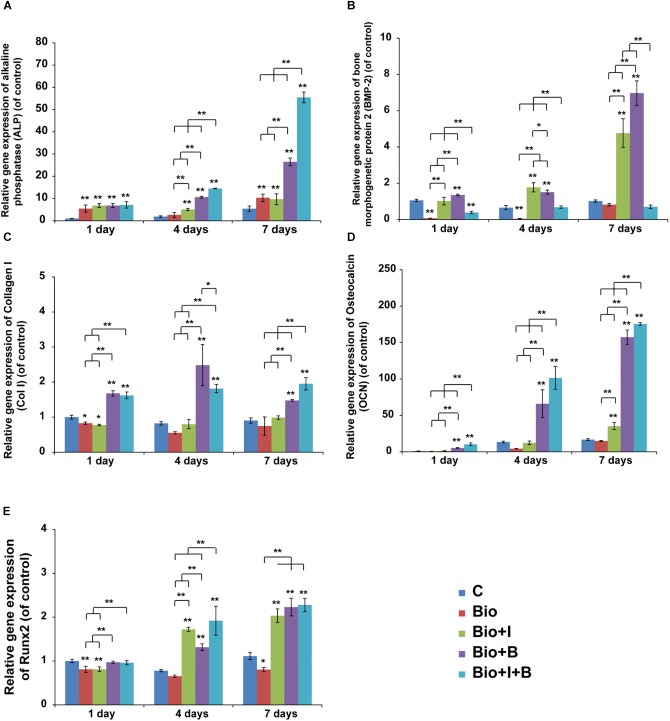
The time course changes in mRNA expression of **(A)** ALP, **(B)** BMP-2 **(C)** Col1, **(D)** OCN, **(E)** Runx2 in MC3T3-E1 on days 1, 4, and 7. Mean values (*n* = 3 samples per group) were represented together with the standard deviation. Error bars denote the standard deviation. ^∗^*P* < 0.05, ^∗∗^*P* < 0.01, ^∗∗^without lines means versus control (C). C, Control; BioCaP, biomimetic calcium phosphate; I, Icariin; B, BMP-2.

From day 1 to day 7, the expression of ALP mRNA was significantly upregulated with the stimulation of BioCaP with Icariin (7, 5, and 10 folds) compared with the control, but was not by BioCaP alone on day 4 (Figure 5A). ALP mRNA expression was dramatically promoted by BioCaP with BMP-2(7, 11 and 26 folds) and BioCaP with Icariin and BMP-2 (7, 14 and 55 folds) on all three time points. BioCaP with both agents significantly increased the ALP expression more than the BioCaP with BMP-2 alone on days 4 and 7 (1.3 and 2.1 folds).

A significant reduction in the expression of BMP-2 mRNA can be detected from day 1 to day 4 when treated with BioCaP alone. Furthermore, BioCaP with Icariin dramatically increased the BMP-2 mRNA expression compared with BioCaP alone on all three times points especially on day 4 (10, 20, and 5 folds) (Figure 5B). Noticeably, the presence of Icariin significantly reduced the expression of BMP-2 mRNA on BioCaP with BMP-2 (−2.5, −2, and −7 folds) after 1, 4, and 7 days’ stimulation.

Unlike the other dramatically upregulated osteogenic mRNA expression, BioCaP with or without Icariin had less ColI expression change after treatment. Addition of Icariin could promote the expression of Col1 mRNA of BioCaP with BMP-2 after 7 days’ treatment (2 folds) (Figure 5C).

BioCaP incorporated with Icariin had significantly higher OCN mRNA expression compared to BioCaP alone on days 4 and 7 (3 and 2.3 folds). With the presence of Icariin, BioCaP with BMP-2 upregulated OCN mRNA expression in comparison with BioCaP with BMP-2 alone on 1, 4 and 7 days (2.0, 1.5, 1.1 folds) (Figure 5D).

BioCaP with or without Icariin did not enhance the expression of Runx2 on day 1 in comparison with the control (Figure 5E). After 4 and 7 days’ stimulation, BioCaP with Icariin significantly increased the Runx2 mRNA expression (2 and 2 folds) when compared with the control and BioCaP alone.BioCaP with both of Icariin and BMP-2 increased the BioCaP with BMP-2 group expression of Runx2 mRNA after 4 days (2 folds) but had no statistical difference after 1 and 7 days treatment (Table 3).

**Table 3:** Factor in mRNA expression as a function of the treatment and of the days after treatment.

mRNA	ALP			BMP-2			Col1			OCN			Runx2		
Time from implant	1 d	4d	7d	1d	4d	7d	1d	4d	7d	1d	4d	7d	1d	4d	7d
Control	1 ± 0.1	2 ± 0.3	5 ± 1.2	1 ± 0.1	1 ± 0.1	1 ± 0.1	1 ± 0.1	1 ± 0.1	1 ± 0.1	1 ± 0.1	13 ± 1.2	17 ± 1.3	1 ± 0.0	1 ± 0.0	1 ± 0.1
BioCaP	6 ± 1.6	3 ± 1.1	10 ± 1.7	0.1 ± 0.0	0.1 ± 0.0	1 ± 0.1	1 ± 0.0	1 ± 0.0	1 ± 0.3	1 ± 0.0	4 ± 0.3	15 ± 0.7	1 ± 0.1	1 ± 0.0	1 ± 0.1
BioCaP + Icariin	7 ± 0.9	5 ± 0.5	10 ± 2.4	1 ± 0.2	2 ± 0.3	5 ± 0.8	1 ± 0.0	1 ± 0.1	1 ± 0.1	1 ± 0.3	12 ± 2.4	35 ± 5.0	1 ± 0.1	2 ± 0.1	2 ± 0.2
BioCaP + BMP-2	7 ± 0.8	11 ± 0.4	26 ± 1.7	1 ± 0.1	2 ± 0.1	7 ± 0.7	2 ± 0.1	2 ± 0.6	1 ± 0.0	5 ± 0.6	66 ± 19.1	157 ± 10.0	1 ± 0.0	1 ± 0.1	2 ± 0.2
BioCaP + Icariin + BMP-2	7 ± 1.4	14 ± 0.2	55 ± 2.4	0.4 ± 0.1	1 ± 0.1	1 ± 0.1	2 ± 0.1	1 ± 0.0	2 ± 0.2	10 ± 1.9	101 ± 15.6	176 ± 1.9	1 ± 0.1	2 ± 0.3	2 ± 0.2

### *In vivo* Experiment

#### Clinical Observations

Twelve weeks after implantation, a total of 30 specimens were harvested. The healing period was uneventful and all the surgical sites healed well without any obvious complications. There were no visual signs of inflammation or any other adverse tissue reactions.

#### Micro-CT Analysis

**FIGURE 6:**
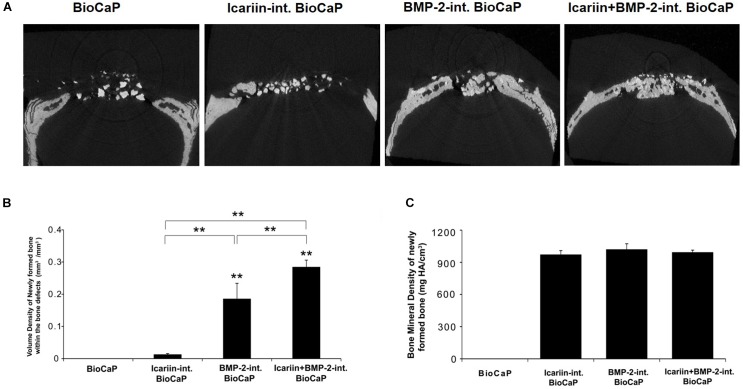
**(A)** The Micro-CT images of the bone defects in scalp of the rats 12 weeks after treatment with BioCaP, Icariin-int. BioCaP, BMP-2-int. BioCaP, and Icariin + BMP-2-int. BioCaP. **(B)** The volume of newly formed bone within the bone defect after 12 weeks postoperation for each group which is analyzed by Micro-CT. Mean value (*n* = 6 specimens per group ) are represented together with the standard deviation. ^∗∗^*P* < 0.01. **(C)** The bone mineral density (mg HA/cm^3^) of the newly formed bone within the bone defect after 12 weeks postoperation for each group which is analyzed by Micro-CT. Mean value (*n* = 6 specimens per group) are represented together with the standard deviation.

The difference between the newly formed bone and BioCap was shown by the analysis software. The BioCaP group had no new bone formation. Micro-CT analysis revealed that the volume density of newly formed bone (mm^3^/mm^3^) in the group with BioCaP granules with an internal depot of BMP-2 and the group with BioCaP granules with an internal depot of Icariin and BMP-2 was significantly higher than the other groups. The group BioCaP with Icariin has a greater volume density of new bone than the group with BioCaP only. Interestingly, the group with both Icariin and BMP-2 had a much higher volume density of new bone than other groups (Figure 6A,B). However, there was no difference in the measured BMD between the groups with BioCaP with Icariin or with BMP-2, or with both. (Figure 6C).

#### Descriptive Light Microscopy

**FIGURE 7:**
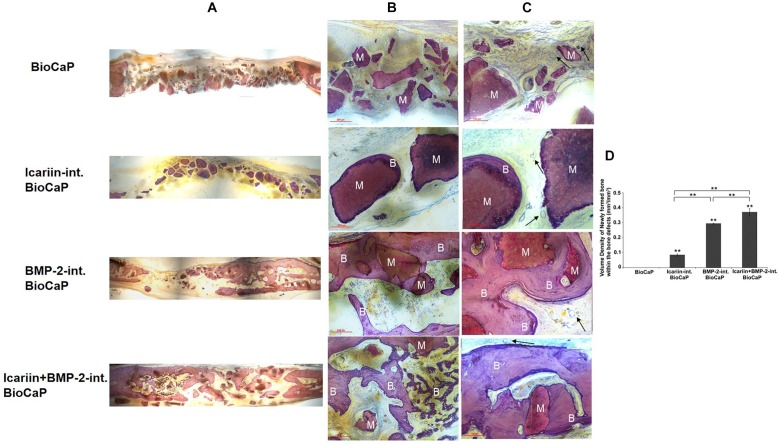
Representative histological micrographs of bone defect of each group at 12 weeks after implantation. The BioCaP granules materials (M) were surround by newly formed bone (B) in Icariin-int. BioCaP, BMP-2-int. BioCaP, and Icariin + BMP-2-int. BioCaP Groups. The slices were surface-stained with McNeal’s Tetrachrome, basic Fuchsine and Toluidine Blue. **(A)** The panoramic images of the defects of four experiment groups. **(B)** Scale bar = 200 μm. **(C)** Scale bar = 100 μm. The vascular (black arrows) was observed in all the groups. **(D)** The volume of newly formed bone within the bone defect after 12 weeks postoperation for each group which is analyzed by histology. Mean value (*n* = 6 specimens per group) are represented together with the standard deviation. ^∗∗^*P* < 0.01

The control group of a critical sized bone defect with no bone substitute confirmed that the defect could not heal 12 weeks after the operation. 12 weeks after the implantation, there was nearly no newly formed bone in the group with BioCaP only. New bone formation can be seen to a greater or lesser extent in the other three groups. In the group BioCaP granules with Icariin new bone only appeared in close contact with the BioCaP granules, in other words, the BioCaP with Icariin was encapsulated with a thin layer of new bone. An interconnected bone network can be clearly observed in the group with BioCaP and BMP-2 and with both groups of Icariin and BMP-2. The BioCaP granules alone were embedded in a quantity of new bone. Interestingly, the group, BioCaP with both Icariin and BMP-2 seems to have more new bone because it covered almost the whole area of the defects, whereas in the group of BioCaP with BMP-2 the new bone covered only a part of the defect (Figure 7A–C).

#### Histomorphometric Results

The quantitative evaluation of the bone formation 12 weeks after implantation is shown in Figure 7D. There was no new bone encapsulating the BioCaP granules alone. The group with BioCaP and both Icariin and BMP-2 produced a significantly higher volume density of new bone than did BioCaP with Icariin or with BMP-2. A greater volume density of bone was observed in the group of BioCaP with BMP-2 than in the group with Icariin.

## Discussion

A previous study showed that the levels of Runx2, BMP-2, and BMP-4 mRNA expression were significantly up-regulated by treating with Icariin. Icariin exerted the osteogenic effect through the induction of Runx2 expression, the production of BMP-2, and the BMP signaling activation. In addition, recent studies have shown that Icariin can modulate the bone formation in human osteoblastic cell line via BMP-2/Smad 4 signal pathway (Zhao et al., 2008; He et al., 2009; Liang et al., 2012). Until now Icariin has commonly been administered orally. Icariin could not maintain an appropriate concentration in bone defects continuously, because of the half life of the Icariin and BMP-2 and because of the way the blood circulation reaches the local tissue in a systemic drug delivery. Previous findings strongly suggest that BioCaP granules make an attractive delivery vehicle for protein (Liu et al., 2013). Therefore, a system for releasing Icariin in a sustained and local manner in bone defects is desperately needed. We have demonstrated in the previous study that adding Icariin significantly increased the osteogenic effects of BMP-2 *in vitro*. In this study, we were eager to estimate the osteogenic potential *in vitro* and *in vivo* of BioCaP alone or BioCaP incorporated with Icariin and/or BMP-2.

Quantitative real-time PCR analysis of MC3T3-E1 cocultured with different payload composite scaffolds was performed to evaluate the expression levels of osteogenic differentiation markers (Figure 5). Interestingly, Icariin with BMP-2 gives a much higher mRNA expression of ALP and OCN than the other combination and it grows rapidly for 7 days. The ALP protein expression is an early marker of changes in osteogenesis. In this study, it was increased by 1.3 and 1.1 fold in 1 and 7 days, respectively, for BioCaP with BMP-2 incorporated with Icariin. In support of this, the mRNA expression of ALP can also upregulated by a factor of 1.3 and 2.1 on days 4 and 7. Adding Icariin to BioCaP with BMP-2 could increase OCN, which is the late marker of differentiation related to the matrix deposition and mineralization, protein expression by a factor of 1.4 on day 7 and mRNA expression by factors of 2.0, 1.5 and 1.1 on 1, 4, and 7 days, respectively.

We have for the first time evaluated using BioCaP incorporated internally with bioactive agents *in vitro*. BioCaP incorporated with or without Icariin did not increase the proliferation of MC3T3-E1 cells. Runx2 controls the osteoblast proliferation and promotes a transition from a proliferative to a post-proliferative stage prior to osteoblast differentiation (Pratap et al., 2003; Galindo et al., 2005). We still need to find out whether the significant up-regulation of Runx2 can partially account for the significant down-regulation of the proliferation of MC3T3-E1 cells. Phimphilai et al. (2006) reported that the transcriptional activity of Runx2 requires BMP signaling and that the sensitivity of cells to BMPs is enhanced by Runx2. In this study, BMP-2 and Runx2 gene expression was upregulated by BioCaP incorporated with Icariin after 4 and 7 days, which is consistent with Phimphilai. The upregulation of BMP-2 gene expression stimulated by BioCaP with Icariin is in line with the speculation in our previous study that Icariin may activate BMP signaling indirectly through extracellular BMP-2 after BMP-2 are produced (Zhang et al., 2014b). However, BioCaP with Icariin often could not increase the osteogenic gene expression on the first day. The reason for this may be that the release rate of Icariin was too slow on the first day, which lead to a very low concentration of Icariin around the MC3T3-E1 cells and consequently it became less effective.

Mineralization is important for bone regeneration. Some hydroxyapatite-based scaffolds are used in bone tissue engineering to provide artificial highly mineralized environments for enhancing osteogenic differentiation and bone regeneration (Zhao et al., 2010). BioCaP granules alone were found to be effective for promoting mineralization on MC3T3-E1 *in vitro*. Icariin alone increased the *in vitro* mineralization (Ma et al., 2011; Zhang et al., 2014b), while the addition of Icariin enhanced the mineralization ability of BioCaP in a way that depends on time as expected. Meanwhile, the presence of Icariin stimulated the mineralization of BioCaP with BMP-2 as expected. These data for the combined effect indicated that Icariin is useful for enhancing mineralization.

The release kinetics is a crucial factor for the osteoinductive efficiency of bioactive agents. The high dose of BMP-2 produced in a burst release is simply adsorbed onto the materials implanted into the bone defect, and results in a low osteoinductive efficiency (Schwarz et al., 2008). In contrast, the internally incorporated BMP-2 showed a gradual, sustained and cell-mediated release of bioactive agents and thus a significantly higher osteoinductive efficiency than when the BMP-2 is adsorbed (Liu et al., 2013). Previous studies have indicated the similarity of the release kinetics of bovine serum albumin (BSA) and BMP-2 (Basmanav et al., 2008; Yilgor et al., 2009). To confirm that the agents were incorporated into BioCaP, two different concentrations of BSA were incorporated into BioCaP tablets to study the protein release in our previous study (Liu et al., 2013). Apart from FITC-BSA (fluorescein-isothiocyanate-BSA) with a concentration of 5.0 μg/ml, BSA labeled with Alexa Fluor^®^ 555 (Alexa-BSA, invitrogen, Carlsbad, CA, United States) was used at a concentration of 0.5 μg/ml. As FITC-BSA release from the samples with 0.5 μg/ml could not be detected, we used in addition Alexa-BSA, which can be detected at lower concentrations than FITC-BSA. A burst release occurred within the first 4 days of incubation, whereas the release occurred at a steady rate after 4th day up to the 16th day. At the 16-day time point, the initial amount of FITC-BSA and Alexa-BSA had been decreased by 55 and 25%, respectively. In another unpublished data, we observed that in the internally-incorporated depot, about 10–14% of BSA release from BioCaP granules in an initial burst release stage (within the first 24 h). Subsequently, BSA was gradually released at a steady rate until the 35th day. Therefore, in this study the agents were incorporated internally into the BioCaP.

Skull defects are favored models for animal and clinical research on bone induction because bone regeneration is either slow or incomplete. The healing of the skull bone is so slow that cranioplasty operations often fail, especially in adult human beings (Takagi and Urist, 1982). The creation of nonunions in animals within the skull was dependent on the size. Defects of a size that will not heal during the lifetime of the animal may be termed critical size defects (Schmitz and Hollinger, 1986). Trephine defects in the vault of the adult cranium of a rat measure 8 mm. (Ray and Holloway, 1957; Takagi and Urist, 1982). The key to establishing critical sized bone defects in the calvarium of a rat is to take great care not to perforate the dura mater. This may cause excessive bleeding that may contribute to the death of the rats (Takagi and Urist, 1982). Critical sized skull defects in adults fail to heal spontaneously, whereas juveniles retain the ability to reossify skull defects of almost any magnitude (Aalami et al., 2004). Thus we selected 8 week old adult SD rats for this study.

The degradability of materials based on CaP is crucial for the *in vivo* longevity and efficacy of their biological effects (Tanuma et al., 2012). The insolubility of the material and the cell-mediated resorption regulated its degradation (Zhang et al., 2012). In the current study, all the BioCaP granules which delivered agents were in close contact with bone or completely encapsulated in the newly formed bone. This finding is consistent with the previous studies (Liu et al., 2013) which found that BioCaP is highly biocompatible. The slow release of BMP-2 plays an important role in bone formation (Wu et al., 2011; Hunziker et al., 2012). In the previous studies, the agents incorporated into the BioCaP granules resulted in a sustained release of agents *in vitro* and BMP-2 delivered by BioCaP granules led to a highly osteoinductive efficiency *in vivo* (Zheng et al., 2013). Only BMP-2 has been used experimentally internally incorporated into the BioCaP granules. Here we have shown for the first time that Icariin, as well as BMP-2, can be delivered and released properly and still have good biocompatibility. The BioCaP granules which were internally incorporated with Icariin, were surrounded by newly formed bone which is evidence that Icariin also can be released sustainably by BioCaP. In our previous *in vitro* experiment, we demonstrated that Icariin alone is a strong osteogenic agent and that the mixture of Icariin and BMP-2 can also enhance significantly the osteogenic activity of BMP-2 (Zhang et al., unpublished). Here we have confirmed it *in vivo*.

Although histological analyses provide unique information on cellularity and dynamic indices of bone remodeling, they have their limitations in assessing the micro-architectures of bone. Histological analyses are derived from a stereological analysis of a few 2D sections, usually with the assumption that the underlying structure is plate like (Parfitt et al., 1987; Parfitt, 1988). On the other hand, micro-CT can measure directly the micro-architecture of bone independently of stereological models (Borah et al., 2001). These landmarks are not always identifiable, which raise questions about their reliability, variability and even subjectivity. The lack of agreement between clinicians in the qualitative study suggests that the assessment of osseous landmarks is somewhat subjective (Norman et al., 2014). In this study, these two approaches were used together to complement their limitations.

We acquired positive results in rats in this study, which may have to be repeated and confirmed in larger species, such as dogs or sheep before human clinical trials can be initiated. Measurements must be made more frequently in addition.

According to our results, we suggest that:

1. In the presence of Icariin, BioCaP have a better osteogenic potential when used to stimulate MC3T3-E1;
2. The addition of Icariin promoted the osteogenic differentiation of MC3T3-E1 when treated with BioCaP incorporated with BMP-2;
3. BioCaP incorporated with Icariin increased the bone formation;
4. The addition of Icariin to BioCaP granules incorporated with BMP-2 significantly enhanced the new bone formation in the critical sized bone defects in SD rats.

## Conclusion

In conclusion, BioCaP incorporated with Icariin can enhance *in vitro* osteogenic differentiation of … cell. BioCaP incorporated with Icariin and BMP-2 showed better osteogenic potential compared with that with BMP-2 alone. Our histological and histomorphometrical findings confirmed our suggestion that Icariin increased the new bone formation in the critical sized bone defects in SD rats when added to BioCaP granules incorporated with BMP-2. The BioCaP granules had good biocompatibility. BioCap granules with Icariin and BMP-2 may be a promising bone substitute for clinical applications.

## Data Availability

All datasets generated for this study are included in the manuscript and/or the supplementary files.

## Author Contributions

XZ wrote the manuscript. XZ, XL, TL, and LD performed the experiments. XZ, YH, and YL conceived or designed the studies. All the authors contributed to analyzing the data.

## Conflict of Interest Statement

The authors declare that the research was conducted in the absence of any commercial or financial relationships that could be construed as a potential conflict of interest.
